# Long-term outcomes after a randomized phase II trial of nerve growth factor eye drops for optic pathway gliomas: four-year follow-up findings in children

**DOI:** 10.1007/s11060-026-05777-z

**Published:** 2026-09-01

**Authors:** Antonio Ruggiero, Giorgio Attinà, Giorgio Placidi, Elena D’Agostino, Alessia Amato, Tommaso Verdolotti, Stefano Mastrangelo, Benedetto Falsini

**Affiliations:** 1 Pediatric Oncology Unit, Fondazione Policlinico Universitario Agostino Gemelli IRCCS, Rome, Italy; 2 Department of Women and Child Health and Public Health, Università Cattolica del Sacro Cuore, Rome, Italy; 3 Ophthalmology, Fondazione Policlinico Universitario A. Gemelli, IRCCS, Rome, Italy; 4 Department of Ophthalmology, Ospedale Pediatrico Bambino Gesu, Rome, Italy; 5 Department of Oncological Radiotherapy and Hematology, Advanced Radiology Center (ARC), Fondazione Policlinico Universitario Agostino Gemelli IRCCS, Rome, Italy

**Keywords:** Nerve growth factor, Optic pathway glioma, Neurofibromatosis type 1, Visual function, Neuroprotection

## Abstract

**Purpose:**

A previous double-blind, randomized, placebo-controlled, phase II clinical trial reported beneficial effects of a short-term treatment (10 days) with murine nerve growth factor (mNGF) eye drops on visual function in children with optic pathway gliomas (OPG). The present study aimed to evaluate long-term changes in clinical and neuroradiological parameters in the cohort of OPG patients who had previously participated in the phase II mNGF trial.

**Methods:**

Fifteen of the 18 patients originally enrolled in the phase II mNGF trial agreed to undergo clinical and neuroradiological monitoring over a 48-month follow-up period. Of these, 9 had originally been randomized to mNGF and 6 to placebo; no additional treatment (mNGF, chemotherapy, or radiotherapy) was administered during the extended follow-up. Every 6 months, patients underwent general clinical and neuro-ophthalmological examination, visual evoked potentials (VEP), and photopic negative response of the electroretinogram (PhNR). Brain MRI was performed every 12 months.

**Results:**

Comparison of initial and final follow-up median values revealed no statistically significant changes in visual acuity, VEP amplitude, PhNR amplitude, or visual field radius. No significant differences were observed in any parameter relative to baseline values of the mNGF trial. Brain MRI demonstrated stable disease in all patients throughout the observation period.

**Conclusion:**

These findings, obtained in an observational extension of the original randomized cohort, indicate favorable long-term safety and tolerability of a short-term course of topical mNGF in children with OPG, with sustained visual and neuroradiological stability over four years, rather than evidence of persistent treatment efficacy. Further prospective, adequately powered and randomized clinical studies are needed to confirm both the short- and long-term clinical efficacy of NGF treatment in preventing OPG-induced visual loss.

## Introduction

Optic pathway gliomas (OPGs) of childhood are low-grade gliomas (LGG) arising anywhere along the optic pathway and account for 3–5% of all pediatric brain tumors. In the pediatric population, OPGs are typically pilocytic astrocytomas (WHO Grade I) characterized by slow progression over many years and an excellent overall survival [1]. Most affected patients are affected by Neurofibromatosis type 1 (NF1), and approximately 15–20% of children with NF1 will develop these tumors during their lifetime [2].

Despite high survival rates, OPGs are associated with significant morbidity. Clinical manifestations depend on tumor location; progressive visual loss is generally regarded as the most common and debilitating consequence in both sporadic and NF1-associated OPGs [3]. Visual impairment can profoundly affect children’s quality of life and neurodevelopment, making preservation of visual function a central goal of therapeutic management [4]. Current treatment modalities — radiotherapy (for children older than 8 years) or chemotherapy regimens (for younger children), as per the SIOP LGG 2004 protocol — are primarily aimed at controlling tumor growth. Notably, no disease-specific treatment is currently available to address OPG-induced visual dysfunction.

Children with OPG often exhibit thinning of the retinal nerve fiber layer (RNFL) due to retinal ganglion cell (RGC) apoptosis, a process closely associated with progressive visual impairment [5]. In NF1-associated OPG, RGC apoptosis may result not only from direct mechanical compression of the optic pathway by the tumor mass but also from a functional deficit caused by the loss of neurofibromin. This loss-of-function leads to hyperactivation of the Ras pathway, phosphorylation of atypical protein kinase C (PKC), and reduction in intracellular cyclic adenosine monophosphate (cAMP) levels [6, 7]. Importantly, preclinical studies have shown that in rodent models RGC apoptosis is preceded by a phase of axonal damage, defining a potential therapeutic window during which intervention could attenuate irreversible cell loss [8, 9]. The work of Toonen et al. identified a clinically relevant time window for future therapeutic strategies aimed at preserving RGC function in NF1-associated OPG [8].

Several preclinical investigations [10–12] and clinical trials in OPG patients [13, 14] have demonstrated the potential of nerve growth factor (NGF), a prototypical neurotrophin, to preserve and protect visual function. NGF mediates its biological effects through two classes of cell-surface receptors: the high-affinity tyrosine kinase receptor TrkA and the low-affinity p75 neurotrophin receptor (p75NTR) [15]. Both receptors are expressed throughout the visual system, from the retina to the visual cortex. In the retina, TrkA and p75NTR are present on RGCs, where endogenous NGF is physiologically produced and serves as a survival factor for these neurons [11].

The potential therapeutic benefit of murine NGF (mNGF) on visual function in OPG was previously assessed in a randomized, double-blind, placebo-controlled phase II clinical trial [14]. Eighteen OPG patients (age range 2–23 years, with and without NF1), presenting with stable disease and severe visual impairment, were randomly assigned to receive either topical mNGF (0.5 mg eye drops administered three times daily for 10 consecutive days; *n* = 10) or a matched placebo formulation (*n* = 8). Outcome measures at baseline and at days 15, 30, 90, and 180 included visual acuity, visual field, VEP, optical coherence tomography (OCT), and the photopic negative response of the electroretinogram (PhNR); MRI was performed at baseline and day 180. Treatment with mNGF led to statistically significant improvements in electrophysiological parameters (PhNR amplitude and VEP) compared with placebo. Additionally, a notable enlargement of the visual field was observed in treated patients in whom reliable measurement was feasible. No severe ocular or systemic adverse events were recorded; mNGF treatment was considered safe and well tolerated. MRI demonstrated no tumor progression in either group [14].

Following completion of the phase II trial, 15 of the 18 patients agreed to undergo an extended off-treatment clinical, functional, and neuroradiological follow-up lasting to 48 months. The primary objective of this extended observation was to evaluate the long-term changes in clinical and neuroradiological parameters in this unique cohort of OPG patients previously exposed to experimental NGF treatment. Here we report the clinical outcomes obtained during the extended follow-up, encompassing visual function results and neuroradiological assessments.

## Methods

### Study population

Fifteen of the 18 patients (or their legal guardians) who had participated in the original mNGF phase II clinical trial provided consent to undergo extended clinical and neuroradiological monitoring for up to 48 months [14]. Of the 15 patients, 9 had originally been randomized to receive mNGF and 6 to receive placebo in the phase II trial; this extended follow-up was therefore an observational study of the original randomized cohort, and no additional mNGF, chemotherapy, or radiotherapy was administered to any patient during the 48-month observation period. Clinical assessments (visual acuity, Goldmann perimetry, VEP, and PhNR) were performed using the same methodology and equipment as in the original phase II trial, with the exception of optical coherence tomography (OCT), which was not repeated during the extended follow-up [14]. Clinical and demographic characteristics of these patients at the time of study entry into the extended follow-up are reported in Table 1. Ten of the 15 patients had severe visual impairment at baseline. The extended follow-up commenced within 6 months after the conclusion of the original mNGF trial.

### Clinical assessments

Every 6 months, patients underwent a comprehensive general clinical and neuro-ophthalmological examination, including measurement of visual acuity (Snellen chart), Goldmann perimetry for visual field assessment, flash visual evoked potentials (VEP), and photopic negative response of the electroretinogram (PhNR). Brain MRI was performed every 12 months. Unlike the original phase II trial, OCT during the extended follow-up was obtained in only a subset of patients rather than in the full cohort; consequently, it was not repeated systematically in all 15 patients and could not be analyzed as a standardized outcome measure. This decision reflected the practical difficulty of obtaining reliable, cooperation-dependent OCT scans in an outpatient setting in a cohort that included young children and patients with NF1-related cognitive comorbidities, for whom reliable image acquisition typically can require sedation [16].

### Neuroradiological assessment

Neuroradiological evaluations were performed by board-certified pediatric neuroradiologists according to the Response Assessment in Neuro-Oncology (RANO) criteria for low-grade glioma [17]. Progressive disease (PD) was defined by any of the following: (1) development of new lesions or detection of abnormal contrast enhancement on follow-up MRI, suggestive of malignant transformation; (2) an increase of ≥ 25% in tumor volume on T2-weighted or fluid-attenuated inversion recovery (FLAIR) sequences; or (3) progressive clinical or visual deterioration not attributable to treatment-related adverse effects. Patients exhibiting tumor volume changes of less than 25% without clinical deterioration were classified as stable disease (SD).

### Statistical analysis

Data are expressed as median values with ranges unless otherwise stated. Statistical comparisons of longitudinal data were performed using the Wilcoxon signed-rank test for non-parametric paired data. Between-group comparisons (mNGF versus placebo) were performed using the Mann–Whitney U test. Exact p-values (two-tailed) are reported for all comparisons, together with the median difference and its 95% bootstrap confidence interval (10,000 resamples) where informative; a p-value < 0.05 was considered statistically significant for all analyses. For visual acuity, non-numeric visual acuity categories (counting fingers, hand motion, light perception) were converted to logMAR equivalents (1.85, 2.3, and 2.7, respectively) prior to statistical analysis, following established conventions [18]. A change in visual acuity of ≥ 0.3 logMAR (approximately 15 ETDRS letters) was considered clinically meaningful, consistent with consensus recommendations for NF1-associated OPG functional outcome measures [19].

## Results

### Patient characteristics

Demographic and clinical characteristics of the 15 enrolled patients are summarized in Table 1.

**Table 1:** Demographic and clinical characteristics of enrolled patients (*N* = 15)

Characteristic	Value
Sex: Male, n (%)	6 (40.0%)
Sex: Female, n (%)	9 (60.0%)
NF1, n (%)	11 (73.3%)
Age at enrolment, median (range), years	12.6 (2.6–24)
Time from diagnosis to study entry, median (range), years	7.1 (1.8–19.6)
Prior biopsy/surgery, n (%)	6 (40.0%)
Prior chemotherapy, n (%)	13 (86.7%)
Assigned to mNGF in phase II trial, n (%)	9 (60%)
Assigned to placebo in phase II trial, n (%)	6 (40%)

NF1, Neurofibromatosis type 1; mNGF, murine nerve growth factor; n, number of patients

Eleven of the 15 patients (73.3%) carried an NF1 diagnosis. The median age at study enrolment was 12.6 years (range: 2.6–24 years). The median time from initial OPG diagnosis to study entry was 7.1 years (range: 1.8–19.6 years). Thirteen patients (86.7%) had previously received chemotherapy; six patients (40%) had undergone prior surgical resection. Nine patients (60%) had originally been randomized to mNGF and six (40%) to placebo in the phase II trial (Table 1); no additional treatment was given during the extended follow-up, so this cohort represents an observational extension of the original randomized comparison rather than a uniformly treated population. Tumor location was classified according to the predominant anatomical site of involvement on MRI: optic nerve only (*n* = 3), optic chiasm (*n* = 10), and retrochiasmal (*n* = 2). Given the frequently large and infiltrative nature of NF1-associated optic pathway gliomas, this classification reflects the tumor’s epicenter rather than a strictly confined lesion; several tumors extended beyond a single anatomical segment (e.g., from the optic nerves into the chiasm, or from the chiasm into the optic tracts), consistent with the pattern of contiguous spread along the optic pathway commonly reported in this population.

### Visual acuity

Visual acuity results for each patient are reported in Table 2. For patients in whom reliable assessment was feasible, initial (baseline) and final follow-up Snellen decimal values are presented for both eyes (30 eyes from 15 patients). Both eyes of each subject were included and analyzed independently, although for statistical purposes the mean value of the two eyes was used in each analysis. Wilcoxon signed-rank analysis revealed no statistically significant change in median visual acuity (logMAR) when comparing baseline and end-of-follow-up values (*p* = 1.000). At the individual level, a clinically meaningful decline (> 3 lines) was observed in the left eye of patient #5, whereas a clinically meaningful improvement (> 3 lines) was noted in the left eye of patient #14; overall, 5 of 30 eyes (16.7%) showed any change in visual acuity between baseline and final follow-up, of which 3 met the pre-specified threshold for clinical meaningfulness (≥ 0.3 logMAR or approximately 15 ETDRS letters).

**Table 2:** Visual acuities (Snellen decimal) recorded at baseline and at the end of follow-up

Patient	Group	Visual Acuity RE (Baseline / End)	Visual Acuity LE (Baseline / End)
1	P	1.0 / 0.7	0.001 / 0.001
2	P	0.04 / 0.04	0.001 / HM
3	P	LP / LP	LP / LP
4	P	0.005 / 0.005	1.0 / 1.0
5	P	HM / HM	0.8 / 0.1 ↓
6	P	0.02 / 0.02	0.05 / 0.05
7	mNGF	HM / HM	0.8 / 0.6
8	mNGF	1.0 / 1.0	1.0 / 1.0
9	mNGF	0.01 / 0.01	0.01 / 0.01
10	mNGF	0.01 / 0.01	0.01 / 0.01
11	mNGF	1.0 / 1.0	1.0 / 1.0
12	mNGF	LP / LP	LP / LP
13	mNGF	LP / LP	LP / LP
14	mNGF	1.0 / 1.0	0.02 / 0.2 ↑
15	mNGF	0.05 / 0.05	0.1 / 0.1

RE, right eye; LE, left eye; P, placebo group; mNGF, treated group; LP, light perception; HM, hand motion; ↑ improvement > 3 Snellen lines; ↓ decline > 3 Snellen lines. Ages are reported at first and last visit

### Visual evoked potentials

**Fig. 1:**
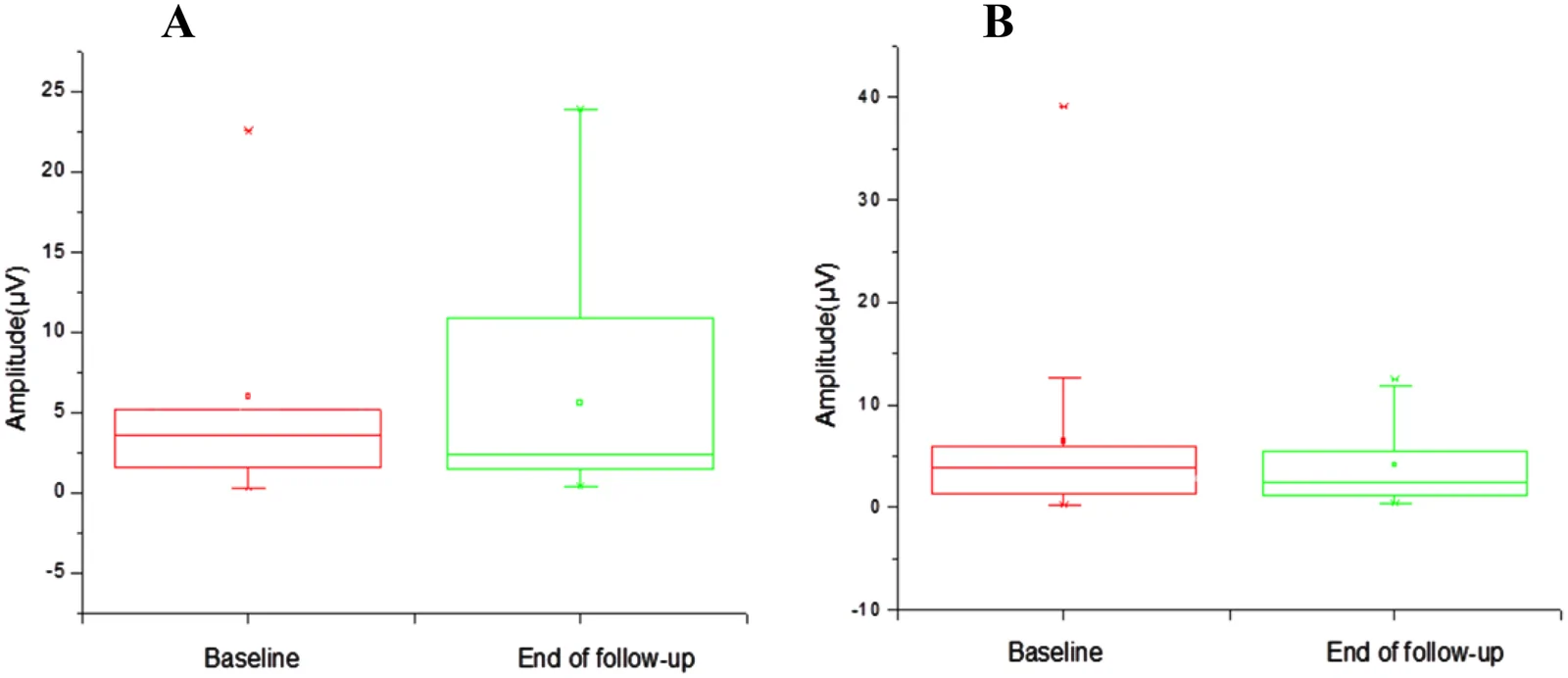
VEP amplitude results are expressed as box plots representing the mean of both eyes for each patient, recorded at baseline and at the end of follow-up. Panel **A** and Panel **B** correspond to the mNGF group and the placebo group, respectively

Flicker VEP amplitude results are illustrated in Fig. 1, presented as box plots of amplitude distribution at baseline and at the end of follow-up, with each data point representing the mean of both eyes for a given patient. No statistically significant change in median VEP amplitude was detected (*p* = 0.685; median difference − 0.26, 95% CI − 1.60 to + 0.97, *n* = 15 patients). VEP phase remained stable throughout the observation period (baseline median: 103°; end-of-follow-up median: 108°).

### Photopic negative response

**Fig. 2:**
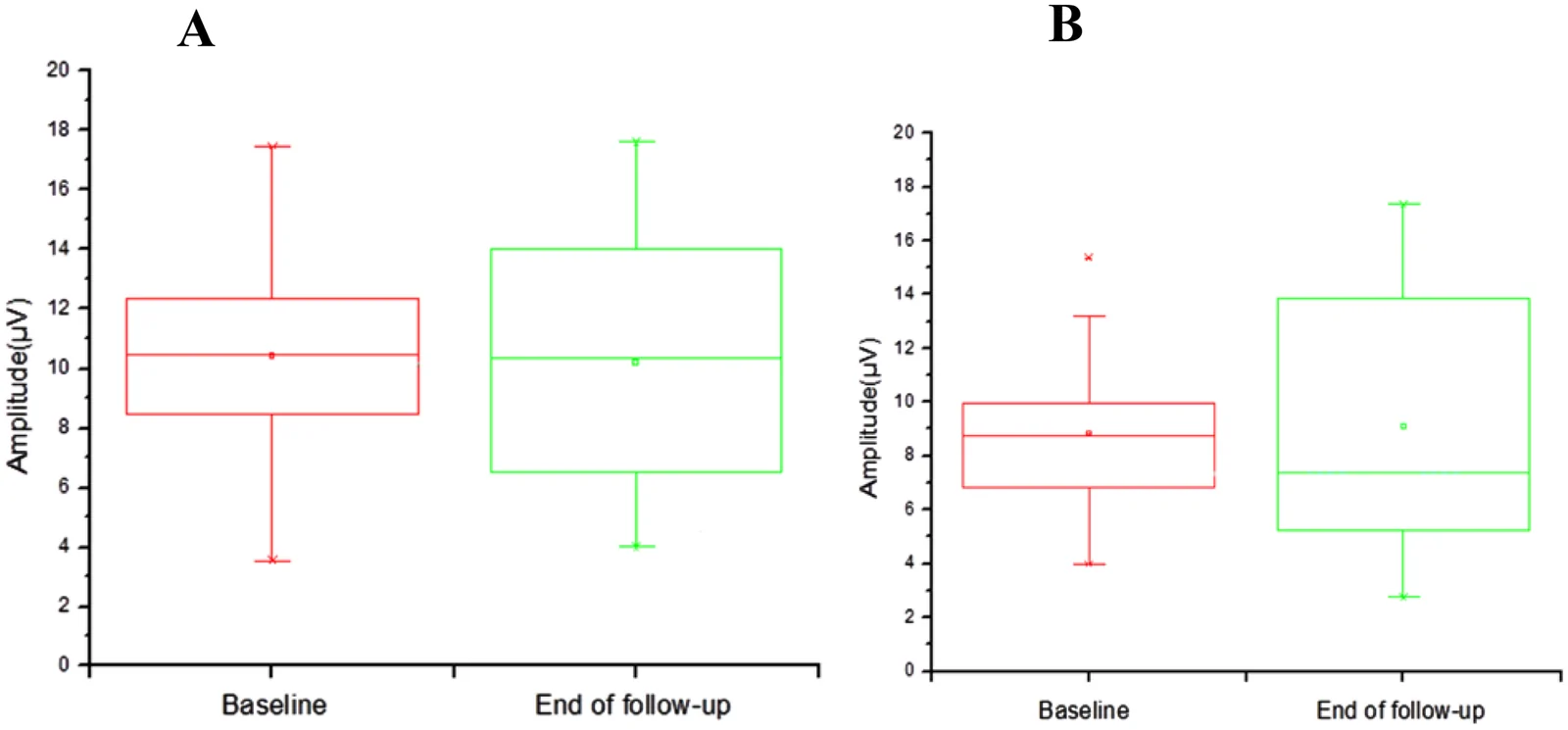
PhNR amplitude, data are expressed as box plots representing the mean of both eyes for each patient, recorded at baseline and at the end of follow-up. Panel **A** and Panel **B** correspond to the mNGF group and the placebo group, respectively

PhNR amplitude data are presented in Fig. 2 as box plots of amplitude distribution, recorded at baseline and at the end of follow-up, with each data point representing the mean of both eyes for a given patient. No statistically significant change in median PhNR amplitude was detected across the follow-up period (*p* = 0.890; median difference − 0.46, 95% CI − 1.71 to + 1.85, *n* = 15 patients). PhNR implicit time likewise showed no significant variation (*p* = 0.330; median difference + 0.30, 95% CI − 0.59 to + 8.64, *n* = 15 patients).

### Visual field

Goldmann visual field results, expressed as the major radius in degrees, are reported in Table 3 for patients in whom reliable perimetric testing was achievable. Values at baseline, at 3 months post-treatment (as previously reported in the phase II trial [14]), and at the end of the extended 48-month follow-up are presented. Wilcoxon signed-rank analysis showed no statistically significant change in median visual field radius when comparing baseline and 48-month values (*p* = 1.000; median difference + 0.2°, 95% CI − 5.9° to + 1.4°, *n* = 15).

**Table 3:** Goldmann visual field results expressed as major radius (degrees) recorded at baseline, 3 months post-treatment, and 48 months post-treatment

Patient	Group	Baseline (deg)	Post-treatment 3 months (deg)	Post-treatment 48 months (deg)
1	P	24.9	18.9	19
2	P	26.5	17.4	17
3	P	15	12	9
4	P	13	12	12
5	P	20	18	11
6	P	11	10	10
7	mNGF	17.3	21.9	18
8	mNGF	12.4	20.6	15
9	mNGF	3.6	20.8	10
10	mNGF	2.8	5.2	6
11	mNGF	10.6	24.4	12
12	mNGF	8.5	20.2	20
13	mNGF	11	16	12
14	mNGF	9.8	11.2	10
15	mNGF	9	13	9

P, placebo group; mNGF, treated group

Re-analysis of the individual visual field data in Table 3 showed that, although the overall group median change from baseline to 48 months was not significant, the change from baseline differed significantly between patients originally assigned to mNGF versus placebo, both at the 3-month post-treatment timepoint (*p* = 0.0004) and, notably, at the 48-month endpoint (*p* = 0.0004): every mNGF-assigned patient showed an increase (or no change) in visual field radius from baseline, whereas every placebo-assigned patient showed a decrease. However, baseline visual field radius was itself larger in the placebo group than in the mNGF group (median 17.5° versus 9.8°; *p* = 0.008), so this divergence may at least partly reflect regression to the mean or a ceiling/floor effect rather than a genuine treatment-related difference, particularly given the known within-visit test-retest variability of Goldmann perimetry, which can approach 20% in some populations, and given that Goldmann perimetry reliability is age-dependent in children [20, 21]. This alternative explanation should be weighed against a true neuroprotective effect when interpreting the early visual field enlargement previously reported in the phase II trial [14]; we did not find evidence that this difference between groups was sustained as a proportional gain over the natural trajectory of each group, and any interpretation of a persistent treatment effect on visual field should be considered hypothesis-generating rather than confirmatory, given the small sample size and non-randomized comparison inherent to this observational extension.

### Neuroradiological outcomes

**Fig. 3:**
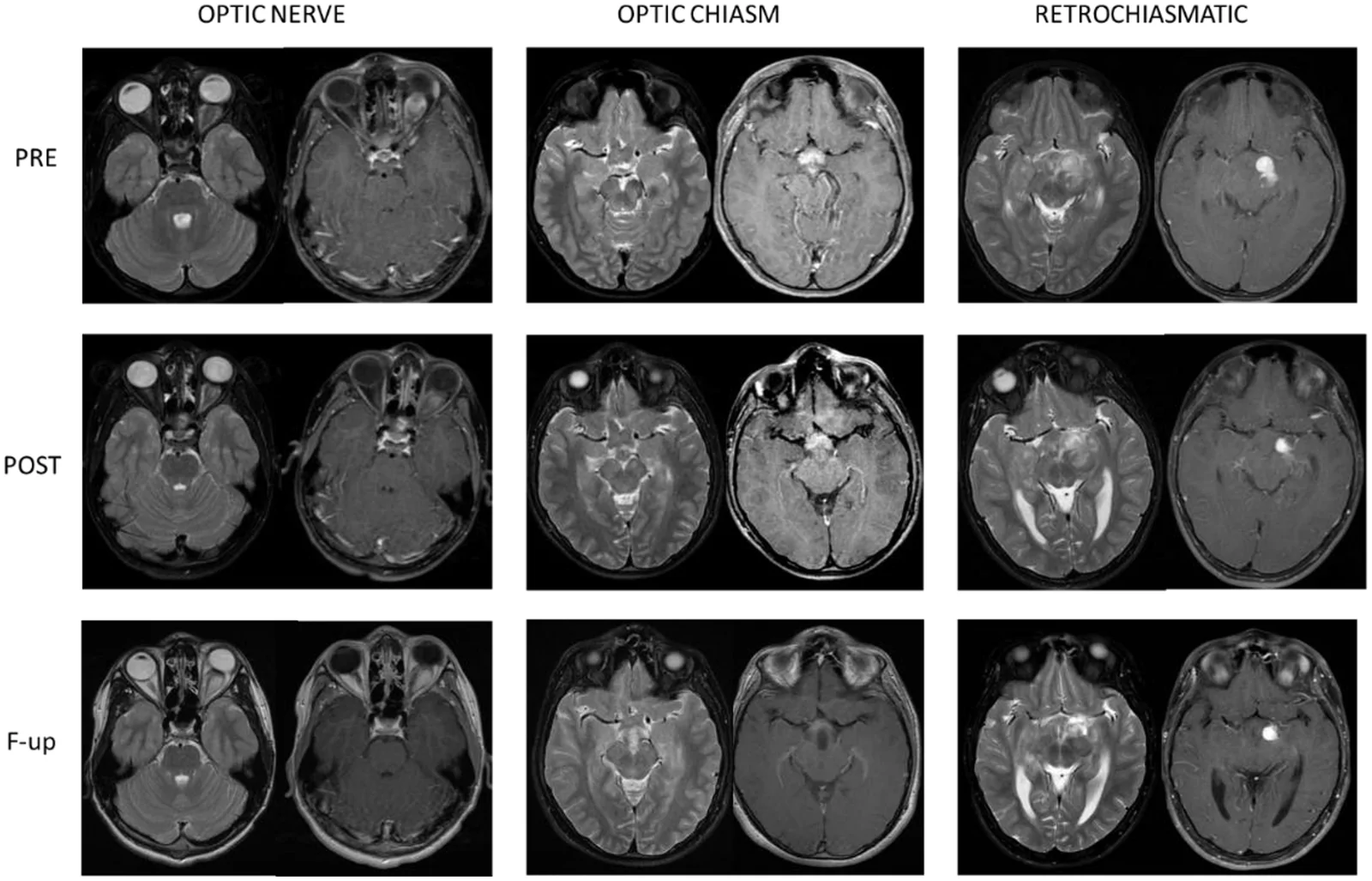
Brain MRI of OPG patients acquired at baseline (upper row), shortly after mNGF administration (middle row), and after four years of follow-up (lower row). For each site of involvement, 2D axial T2w Fast Spin Echo (FSE) sequence (left image) and 2D axial T1w FSE after contrast administration sequence (right image) are shown

Brain MRI showed no significant change in tumor volume throughout the extended follow-up in any of the enrolled patients. Two patients (patients #5 and #8) exhibited a minor volumetric increase of approximately 10% on follow-up imaging; however, these changes did not meet the RANO criteria for progressive disease and were classified as stable disease. Accordingly, stable disease was confirmed in all 15 patients throughout the four-year observation period. Figure 3 displays representative MRI scans acquired at baseline (upper row), shortly after mNGF administration (middle row), and after four years of follow-up (lower row), illustrating different sites of optic pathway involvement: optic nerve (left column), optic chiasm (middle column), and retrochiasmatic region (right column). For each case, both enhancing and non-enhancing tumor components were evaluated. Stable disease was confirmed in all cases at the four-year follow-up.

### Ocular safety

No off-target ocular adverse events were observed during the 4-year extended follow-up. Slit-lamp examination performed at each follow-up visit did not reveal corneal sensitivity changes, corneal or lens opacities, or eyelid abnormalities in any of the 9 patients exposed to mNGF.

## Discussion

The present study reports the four-year clinical and neuroradiological outcomes of children with OPG who had previously participated in a randomized phase II trial of topical mNGF versus placebo, and who received no additional treatment during this extended, observational follow-up period. The principal findings indicate overall stability of the assessed visual function parameters, including visual acuity, VEP amplitude, PhNR amplitude, and visual field radius, as well as stable neuroradiological status, over an extended observation period, with no significant differences between the original mNGF and placebo groups other than a divergence in visual field change and may reflect regression to the mean rather than a treatment effect. These results collectively support the favorable long-term safety profile of topical NGF in pediatric OPG; because this extended phase was observational and non-randomized, however, they should not be interpreted as evidence of sustained treatment efficacy.

The neuroprotective properties of exogenous NGF on RGC viability have been well established in preclinical models. Administration of mNGF has been shown to attenuate RGC apoptosis following optic nerve transection and ocular ischemia [10, 22, 23]. The molecular basis of this neuroprotective effect likely involves NGF–TrkA signaling, which upregulates the anti-apoptotic mediator BCL-2 and suppresses caspase activation [24]. In support of this mechanism, Sivilia et al. demonstrated that a single intravitreal NGF injection in a rat model of permanent carotid artery occlusion prevented RGC degeneration by modulating the Bax/BCL-2 ratio and c-Jun expression [23].

More recent preclinical studies using optic nerve crush (ONC) rat models have extended these findings, demonstrating that both intravitreal recombinant human NGF (rhNGF) and rhNGF eye drop formulations can promote axonal regrowth and reduce RGC loss. The proposed mechanism involves interference with apoptotic pathways, including inhibition of the NogoA/p75NTR complex, suppression of Rho/ROCK2 signaling, and modulation of axonal growth-inhibitory pathways [12].

A potential concern regarding the therapeutic use of NGF in OPG relates to the theoretical risk of pro-proliferative effects on neoplastic glial tissue. However, converging in vitro and in vivo evidence suggests that this risk is low in the context of pediatric pilocytic astrocytomas. These tumors are characterized by downregulation of TrkA — the receptor whose activation is associated with neoplastic cell proliferation and survival — and by upregulation of the pro-apoptotic p75NTR receptor [25]. This receptor profile may partly explain the characteristically indolent behavior of LGGs and may even support a differentiating role of NGF on LGG cells. Indeed, in vitro studies have demonstrated that NGF inhibits rat C6 glioma cell proliferation and promotes cellular differentiation, evidenced by morphological changes, cellular hypertrophy, and growth cone formation [26], findings subsequently confirmed in vivo [27]. More recently, Meco et al. showed that NGF treatment prevents proliferation of pediatric LGG cell lines through induction of cellular senescence [28].

The stable MRI findings observed across the four-year follow-up are consistent with the established natural history of pediatric pilocytic astrocytomas, which typically exhibit prolonged periods of neuroradiological quiescence [1]. Importantly, the two patients who displayed minor volumetric increases (approximately 10%) did not meet RANO criteria for progressive disease, and neither experienced significant clinical deterioration, reinforcing the classification of stable disease for all cases. It should be emphasized that this indolent natural history, rather than a specific effect of mNGF, is a plausible explanation for the neuroradiological and visual stability observed in both the mNGF and placebo subgroups of the present cohort; the similar long-term outcomes in patients originally assigned to placebo support this interpretation and argue against attributing the observed stability primarily to the initial mNGF course.

Several limitations of this study warrant acknowledgment. The cohort size was small (*n* = 15; 9 originally assigned to mNGF and 6 to placebo), reflecting the rarity of the condition and the pragmatic constraints of recruiting from a completed phase II trial; this limited statistical power, particularly for between-group comparisons, and increases the risk that chance imbalances between the two small subgroups (as observed for baseline visual field radius) could be misinterpreted as treatment effects. No new control group was established for this extended follow-up, and the absence of randomization in the observational phase precludes definitive inferences regarding treatment-specific long-term effects. Furthermore, because visual acuity and visual field data include both eyes of most patients, individual eyes are not fully statistically independent observations, which was not accounted for in the primary analyses and represents an additional source of uncertainty around the reported p-values. Additionally, OCT, a sensitive marker of RNFL thickness and RGC integrity, was not systematically included in the extended follow-up protocol, representing a missed opportunity for structural-functional correlation.

In conclusion, this extended four-year, observational follow-up of the original randomized cohort of children with OPG demonstrates the sustained safety and favorable tolerability of a short-term course of topical mNGF, with no clear evidence, from this non-randomized extension, of persistent superiority over placebo in visual or neuroradiological outcomes. The observed neuroradiological and functional stability over time provides reassurance regarding the absence of long-term adverse effects, including tumor stimulation, associated with this treatment. Future prospective, adequately powered and randomized clinical studies with standardized outcome measures will be necessary to conclusively establish the short- and long-term clinical efficacy of NGF therapy in preventing OPG-induced visual loss.

## Data Availability

No datasets were generated or analysed during the current study.

## References

[1] Rasool N, Odel JG, Kazim M (2017) Optic pathway glioma of childhood. Curr Opin Ophthalmol 28(3):289–295. 10.1097/ICU.0000000000000370 28257299

[2] Khatua S, Gutmann DH, Packer RJ (2018) Neurofibromatosis type 1 and optic pathway glioma: molecular interplay and therapeutic insights. Pediatr Blood Cancer 65(3):e26838. 10.1002/pbc.26838 29049847

[3] Fried I, Tabori U, Tihan T, Reginald A, Bouffet E (2013) Optic pathway gliomas: a review. CNS Oncol 2(2):143–159. 10.2217/cns.12.47 PMC6169473 25057976

[4] Sturgess B, Brown M, Fraser F, Bailey S (2018) A qualitative study of the multi-professional team approach to the management of children with optic pathway gliomas. Pediatr Blood Cancer 65(12):e27377. 10.1002/pbc.27377 30084225

[5] Avery RA, Cnaan A, Schuman JS et al (2015) Longitudinal change of circumpapillary retinal nerve fiber layer thickness in children with optic pathway gliomas. Am J Ophthalmol 160(5):944–952e1. 10.1016/j.ajo.2015.07.036 PMC4661093 26231306

[6] Milde T, Rodriguez FJ, Barnholtz-Sloan JS et al (2021) Reimagining pilocytic astrocytomas in the context of pediatric low-grade gliomas. Neuro Oncol 23(10):1634–1646. 10.1093/neuonc/noab138 PMC8485452 34131743

[7] Gutmann DH, Parada LF, Silva AJ, Ratner N (2012) Neurofibromatosis type 1: modeling CNS dysfunction. J Neurosci 32(41):14087–14093. 10.1523/JNEUROSCI.3242-12.2012 PMC3477849 23055477

[8] Toonen JA, Ma Y, Gutmann DH (2017) Defining the temporal course of murine neurofibromatosis-1 optic gliomagenesis reveals a therapeutic window to attenuate retinal dysfunction. Neuro Oncol 19(6):808–819. 10.1093/neuonc/now267 PMC5464459 28039362

[9] Porciatti V, Ventura LM (2012) Retinal ganglion cell functional plasticity and optic neuropathy: a comprehensive model. J Neuroophthalmol 32(4):354–358. 10.1097/WNO.0b013e3182745600 PMC3694715 23196946

[10] Carmignoto G, Maffei L, Candeo P, Canella R, Comelli C (1989) Effect of NGF on the survival of rat retinal ganglion cells following optic nerve section. J Neurosci 9(4):1263–1272. 10.1523/JNEUROSCI.09-04-01263.1989 PMC6569868 2467970

[11] Micera A, Lambiase A, Aloe L et al (2004) Nerve growth factor involvement in the visual system: implications in allergic and neurodegenerative diseases. Cytokine Growth Factor Rev 15(6):411–417. 10.1016/j.cytogfr.2004.09.003 15561599

[12] Mesentier-Louro LA, Rosso P, Carito V et al (2019) Nerve growth factor role on retinal ganglion cell survival and axon regrowth: effects of ocular administration in experimental models of optic nerve injury. Mol Neurobiol 56(2):1056–1069. 10.1007/s12035-018-1154-1 29869196

[13] Falsini B, Chiaretti A, Barone G et al (2011) Topical nerve growth factor as a visual rescue strategy in pediatric optic gliomas: a pilot study including electrophysiology. Neurorehabil Neural Repair 25(6):512–520. 10.1177/1545968310397201 21444653

[14] Falsini B, Chiaretti A, Rizzo D et al (2016) Nerve growth factor improves visual loss in childhood optic gliomas: a randomized, double-blind, phase II clinical trial. Brain 139(Pt 2):404–414. 10.1093/brain/awv366 26767384

[15] Huang EJ, Reichardt LF (2001) Neurotrophins: roles in neuronal development and function. Annu Rev Neurosci 24:677–736. 10.1146/annurev.neuro.24.1.677 PMC2758233 11520916

[16] Avery RA, Hwang EI, Ishikawa H et al (2014) Handheld optical coherence tomography during sedation in young children with optic pathway gliomas. JAMA Ophthalmol 132(3):265–271. 10.1001/jamaophthalmol.2013.7649 PMC4445404 24435762

[17] van den Bent MJ, Wefel JS, Schiff D et al (2011) Response assessment in neuro-oncology (RANO): assessment of outcome in trials of diffuse low-grade gliomas. Lancet Oncol 12:583–593 10.1016/S1470-2045(11)70057-2 21474379

[18] Schulze-Bonsel K, Feltgen N, Burau H, Hansen L, Bach M (2006) Visual acuities hand motion and counting fingers can be quantified with the Freiburg Visual Acuity Test. Invest Ophthalmol Vis Sci 47(3):1236–1240 10.1167/iovs.05-0981 16505064

[19] Fisher MJ, Avery RA, Allen JC et al (2013) Functional outcome measures for NF1-associated optic pathway glioma clinical trials. Neurology 81(21 Suppl 1):S15–S24. 10.1212/01.wnl.0000435745.95155.b8 PMC3908337 24249802

[20] Patel DE, Cumberland PM, Walters BC, Russell-Eggitt I, Rahi JS, OPTIC study group (2015) Study of Optimal Perimetric Testing in Children (OPTIC): feasibility, reliability and repeatability of perimetry in children. PLoS ONE 10(6):e0130895. 10.1371/journal.pone.0130895 PMC4474916 26091102

[21] Bittner AK, Iftikhar MH, Dagnelie G (2011) Test-retest, within-visit variability of Goldmann visual fields in retinitis pigmentosa. Invest Ophthalmol Vis Sci 52(11):8042–8046. 10.1167/iovs.11-8321 PMC3208001 21896857

[22] Carmignoto G, Comelli MC, Candeo P et al (1991) Expression of NGF receptor and NGF receptor mRNA in the developing and adult rat retina. Exp Neurol 111(3):302–311. 10.1016/0014-4886(91)90097-v 1847878

[23] Sivilia S, Giuliani A, Fernández M et al (2009) Intravitreal NGF administration counteracts retina degeneration after permanent carotid artery occlusion in rat. BMC Neurosci 10:52. 10.1186/1471-2202-10-52 PMC2699342 19473529

[24] Coassin M, Lambiase A, Sposato V et al (2008) Retinal p75 and Bax overexpression is associated with retinal ganglion cell apoptosis in a rat model of glaucoma. Graefes Arch Clin Exp Ophthalmol 246(12):1743–1749. 10.1007/s00417-008-0913-5 18751719

[25] Chiaretti A, Aloe L, Antonelli A et al (2004) Neurotrophic factor expression in childhood low-grade astrocytomas and ependymomas. Childs Nerv Syst 20(6):412–419. 10.1007/s00381-004-0959-6 15138791

[26] Watanabe T, Katayama Y, Kimura S, Yoshino A (1999) Control of proliferation and survival of C6 glioma cells with modification of the nerve growth factor autocrine system. J Neurooncol 41(2):121–128. 10.1023/a:1006127624487 10222432

[27] Kimura S, Yoshino A, Katayama Y, Watanabe T, Fukushima T (2002) Growth control of C6 glioma in vivo by nerve growth factor. J Neurooncol 59(3):199–205. 10.1023/a:1019919019497 12241115

[28] Meco D, Di Francesco AM, Melotti L, Ruggiero A, Riccardi R (2019) Ectopic nerve growth factor prevents proliferation in glioma cells by senescence induction. J Cell Physiol 234(5):6820–6830. 10.1002/jcp.27430 30417351

